# Self-powered broadband photodetector based on MoS_2_/Sb_2_Te_3_ heterojunctions: a promising approach for highly sensitive detection

**DOI:** 10.1515/nanoph-2022-0413

**Published:** 2022-10-28

**Authors:** Hao Wang, Yaliang Gui, Chaobo Dong, Salem Altaleb, Behrouz Movahhed Nouri, Martin Thomaschewski, Hamed Dalir, Volker J. Sorger

**Affiliations:** Department of Electrical & Computer Engineering, The George Washington University, 800 22nd Street NW 5000 Science & Engineering Hall, Washington, DC 20052, USA; Optelligence LLC, 10703 Marlboro Pike, Upper Marlboro, MD 20772, USA

**Keywords:** 2D material, broadband, photodetector, self-powered

## Abstract

Topological insulators have shown great potential for future optoelectronic technology due to their extraordinary optical and electrical properties. Photodetectors, as one of the most widely used optoelectronic devices, are crucial for sensing, imaging, communication, and optical computing systems to convert optical signals to electrical signals. Here we experimentally show a novel combination of topological insulators (TIs) and transition metal chalcogenides (TMDs) based self-powered photodetectors with ultra-low dark current and high sensitivity. The photodetector formed by a MoS_2_/Sb_2_Te_3_ heterogeneous junction exhibits a low dark current of 2.4 pA at zero bias and 1.2 nA at 1V. It shows a high photoresponsivity of >150 mA W^−1^ at zero bias and rectification of 3 times at an externally applied bias voltage of 1V. The excellent performance of the proposed photodetector with its innovative material combination of TMDs and TIs paves the way for the development of novel high-performance optoelectronic devices. The TIs/TMDs transfer used to form the heterojunction is simple to incorporate into on-chip waveguide systems, enabling future applications on highly integrated photonic circuits.

## 1 Introduction

Transition metal chalcogenides (TMDs) have been widely explored as active materials for high-performance optoelectronics due to their unique physical properties [1–3]. Transition metal chalcogenides optoelectronics usually suffers from surface oxidation, high contact resistance, and relatively low mobilities. To alleviate the aforementioned disadvantages of TMD-based photodetectors, recent technologies utilize plasmonic enhancement to improve the light–matter interaction of TMDs, while others use heterostructures to adjust inherent electrical and optical characteristics and graphene contact to aid charge carrier injection and extraction; those all exhibit attractive performance improvements such as higher sensitivity, broader frequency response, improved external quantum efficiencies (EQE), higher detectivity (D*), and a broader spectral photoresponse [4–8]. However, combining topological insulators (TIs) as a novel quantum materials group with the attractive features of TMDs has remained largely unexplored. Topological insulators themselves exhibit various appealing optoelectronic features [9–11], which find applications in high-performance photodetection, e.g., due to their unique energy band structure. Furthermore, the topologically protected gapless conductive edge states or surface states in TIs offer extraordinarily high mobility and a broad detection spectrum compared to graphene, which has zero bandgaps [12–16]. Topological insulators are also mechanically flexible materials that can be used in wearable technology. Most TMDs are easily oxidized under atmospheric environments, whereas TIs are more stable in normal conditions. As a monolayer, molybdenum disulfide (MoS_2_) has a direct bandgap of 1.8 eV, whereas, in bulk, it has an indirect bandgap of 1.3 eV [17]. The visible to the infrared absorption spectrum of MoS_2_ ranges from 350 to 950 nm, which is utilized in various optoelectronic applications such as light harvesting, photovoltaics, and photodetection [18–23]. Heterojunctions are widely applied to self-powered photodetectors since the built-in electric field can efficiently separate the electron-hole pairs to enhance the photoresponsivity and speed under zero bias voltage. Different combinations of materials or technics have been demonstrated to improve the performance of the optoelectronic devices [24–28]. Recently, a tellurium at selenium roll-to-roll nanotube heterojunction and cadmium sulfide at cadmium selenide core/shell quantum dots showed the enhanced capacity for self-powered broadband photodetection but also significantly improved photocurrent density and stability in different environments [29, 30]. Here, we demonstrate a vertical heterojunction-based photodetector with TIs and TMDs, which provide a promising tool to bring different electrical and optical properties by creating the junction compared to the individual materials. The heterojunction-based photodetector, formed by MoS_2_ and Sb_2_Te_3_ exfoliation and deterministic transfer to a silicon oxide substrate, shows reduced dark current, high responsivity *R* = *I*_photo_/*P*_laser_ (defined by the ratio between the photocurrent *I*_photo_ and the incident laser power *P*_laser_), and a self-driven detection range from visible to near-infrared wavelengths. The photocarrier separation is accelerated, and photocarrier recombination is suppressed due to the inherent difference in their energy level. Compared to single TMDs, it also exhibits a reduced dark current, which is beneficial for achieving high detection sensitivities at reduced power consumption. The dark current is ∼10 pA, and the light-induced on/off ratio is more than 10^3^ due to the interlayer built-in field and a broadband response from 500 to 900 nm. Besides the demonstrated free-space application, the proposed device has potential in densely integrated applications such as digital to analog converter systems or photonic integrated circuits due to the ease of integration with photonic waveguide technology [31–33].

## 2 Results and discussion

**Figure 1:**
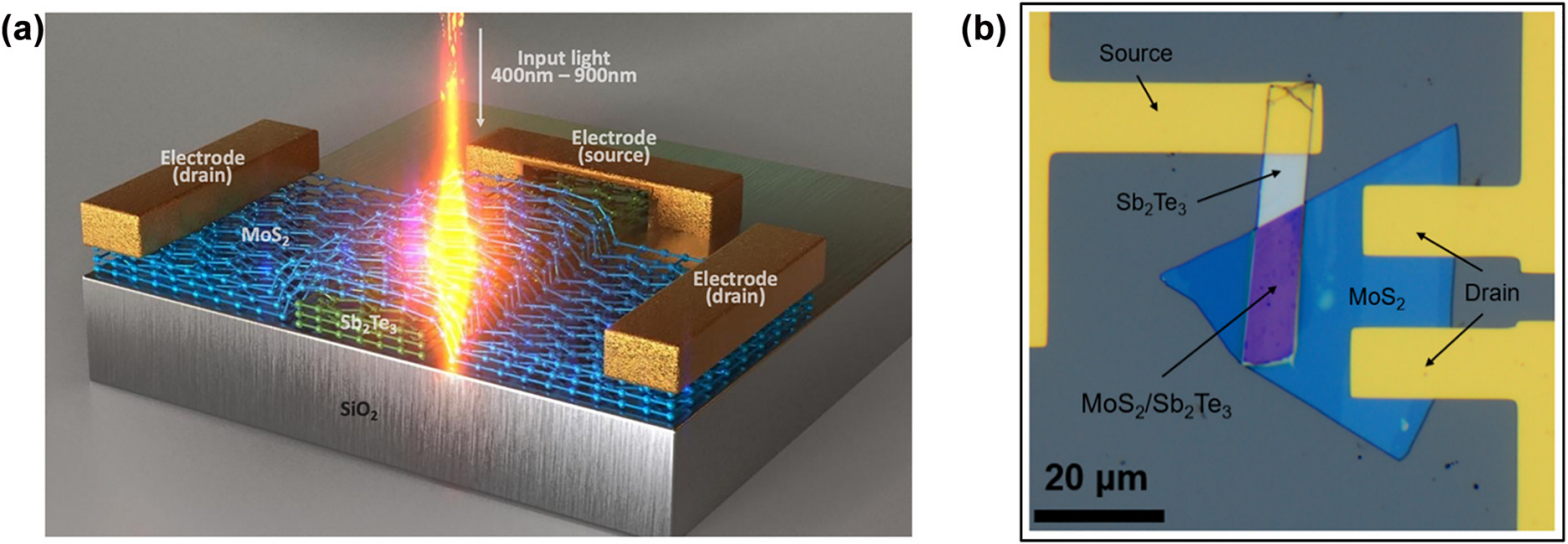
Sb_2_Te_3_/MoS_2_ pn junction heterostructure photodetector. (a) A schematic representation of the Sb_2_Te_3_/MoS_2_ van der Waals p-n junction photodetector. (b) The optical microscope image of PN junction device.

**Figure 2:**
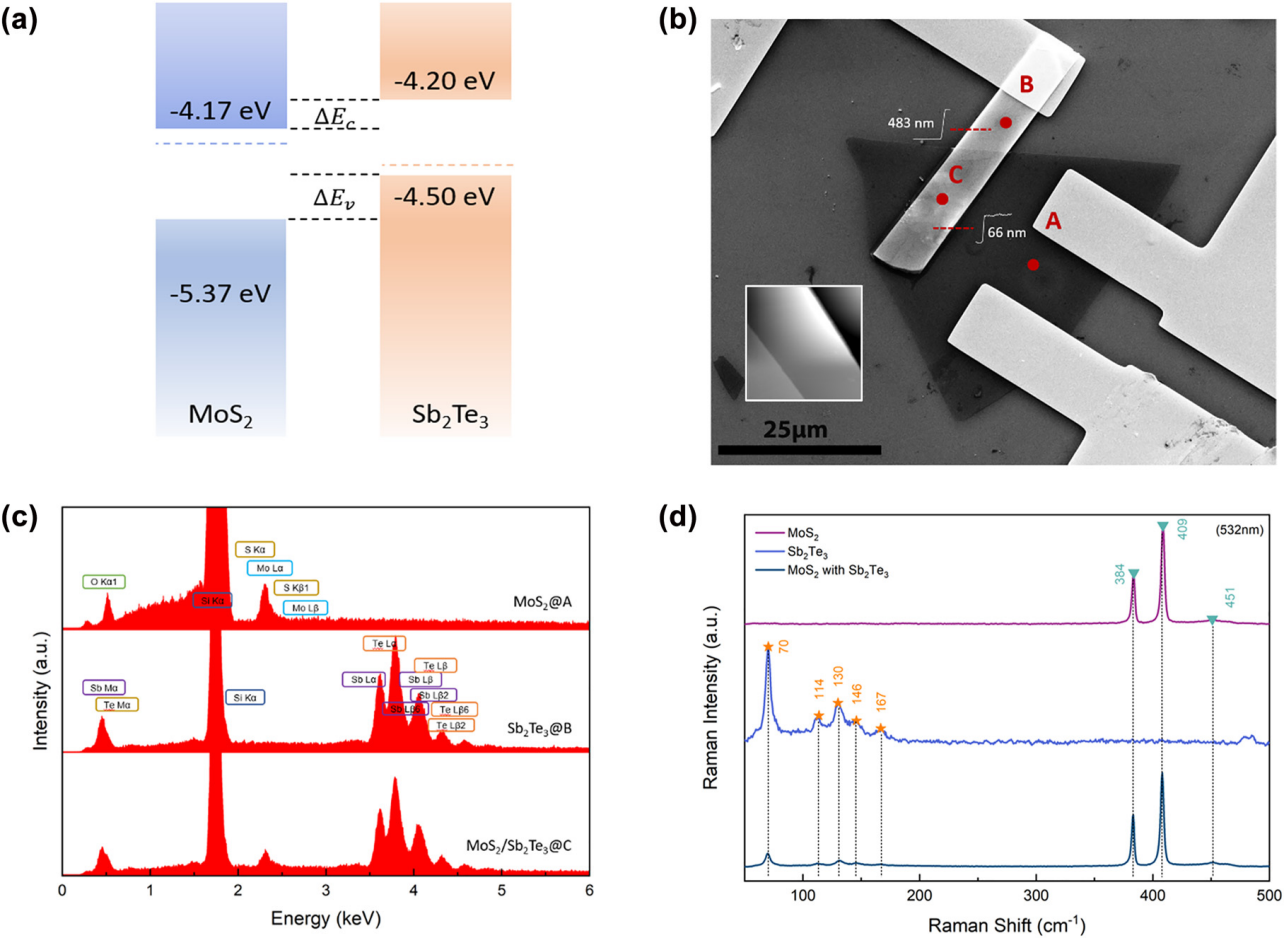
Material characterization of the Sb2Te3/MoS2 heterojunction. (a) Band structures of the vdW layered MoS_2_/Sb_2_Te_3_ heterojunction (b) SEM image of the fabricated device with point A on Sb_2_Te_3_, point B on MoS_2_ and point C on the MoS_2_/Sb_2_Te_3_ heterojunction. Two electrodes are on top of the MoS_2_ layer and one on top of the Sb_2_Te_3_. (c) As shown in the SEM image, EDS signals were collected at three distinct positions to characterize the materials. (d) Raman spectra were collected from the pure MoS_2_ and Sb2Te_3_ and the Sb2Te3/MoS2 PN junction region with a 532 nm laser.

To fabricate the device, Sb_2_Te_3_ and MoS_2_ layers were prepared by mechanical exfoliation and transferred to a SiO_2_/Si substrate by the pick and drop technique. These two layers are stacked by weak van der Waals force (Figure 1a). Three Ti/Au electrodes formed by electron beam evaporation act as the electrical contact pads to inject and extract the photocurrent generated by the photodetector. Two electrodes are placed on the top of the MoS_2_, and one electrode is placed on the Sb_2_Te_3_ (Figure 1b). The compact total junction size is measured to be only 160 μm^2^. A Schottky barrier is present when the electrode is contacted with the MoS_2_/Sb_2_Te_3_ layers to reach the equilibrium state, and it also generates a tunneling current. The Schottky barrier height is strongly dependent on the metal work function. However, the applied heterostructure minimizes the depletion region due to the atomically thin layer. The photocarriers can be efficiently separated at the heterostructure interface with the built-in electric field in the PN heterojunction or an external electric field from an applied bias, yielding photocurrent across the heterostructure channel. As a result, there is no significant potential barrier in the forward direction, which increases the photodetector’s responsivity (Figure 2a).

Raman and energy-dispersive X-ray spectroscopy (EDS) measurements are used to characterize the spatial structure and properties of the vertically stacked MoS_2_/Sb_2_Te_3_ system. Raman spectra are collected via 532 nm laser illumination. The Raman signal of MoS_2_ showed two distinct peaks at 384 cm^−1^ and 409 cm^−1^ in the spectrum Figure 2d, which indicated the in-plane Mo-S phonon mode (E^1^_2g_ at 384 cm^−1^) and out-of-plane Mo-S phonon mode (A_1g_ at 409 cm^−1^) of MoS_2_, respectively [34, 35]. Raman Spectra evidence the MoS_2_ multilayer film with a stoke shift of 25 cm^−1^ [36]. The Raman spectrum of Sb_2_Te_3_ consists of different peaks related to Sb_2_Te_3_ vibrations: 70 cm^−1^, 114 cm^−1^, and 130 cm^−1^ with two minor peaks centered at 97 cm^−1^, and 105 cm^−1^ between, and 146 cm^−1^, 167 cm^−1^. The 70 cm^−1^, 97 cm^−1^, and 167 cm^−1^ peaks indicated the A_1g_ and E_g_ normal modes of the Sb–Te vibrations. The 114 cm^−1^, 105 cm^−1^, and 146 cm^−1^ indicate the Te–Te interactions, which together consist of the whole structure of Sb_2_Te_3_ [36]. The thickness of Sb_2_Te_3_ is estimated to be above 65 nm [37]. Energy dispersive spectroscopy (EDS) is employed to explore the element information collected from the single layers (the exfoliated MoS_2_ (point C), and Sb_2_Te_3_ (point A) and stacked layers (MoS_2_/Sb_2_Te_3_ [point B]) on the device, respectively (Figure 2b). The corresponding MoS_2_ EDS spectrum shows the *L* series peaks of Mo and S K_α_ peak is very pronounced; therefore, only a widened peak with a tail towards the high energy end can be seen with EDS. The elemental compositions (Sb/Te) of the exfoliated Sb_2_Te_3_ layers were also determined by EDS. We observed two weak X-ray emission peaks corresponding to Sb and Te. The spectrum also showed the characteristic peaks of Si, which originates from the used substrate. The absence of any other peaks indicates that the Sb_2_Te_3_ layers are formed from Sb and Te, only. The quantitative atomic ratio of Sb and Te is roughly 38.7–61.3%, which is near to the 2:3 stoichiometry ratio in accordance with the EDS investigations of Sb_2_Te_3_ [38–40].

**Figure 3:**
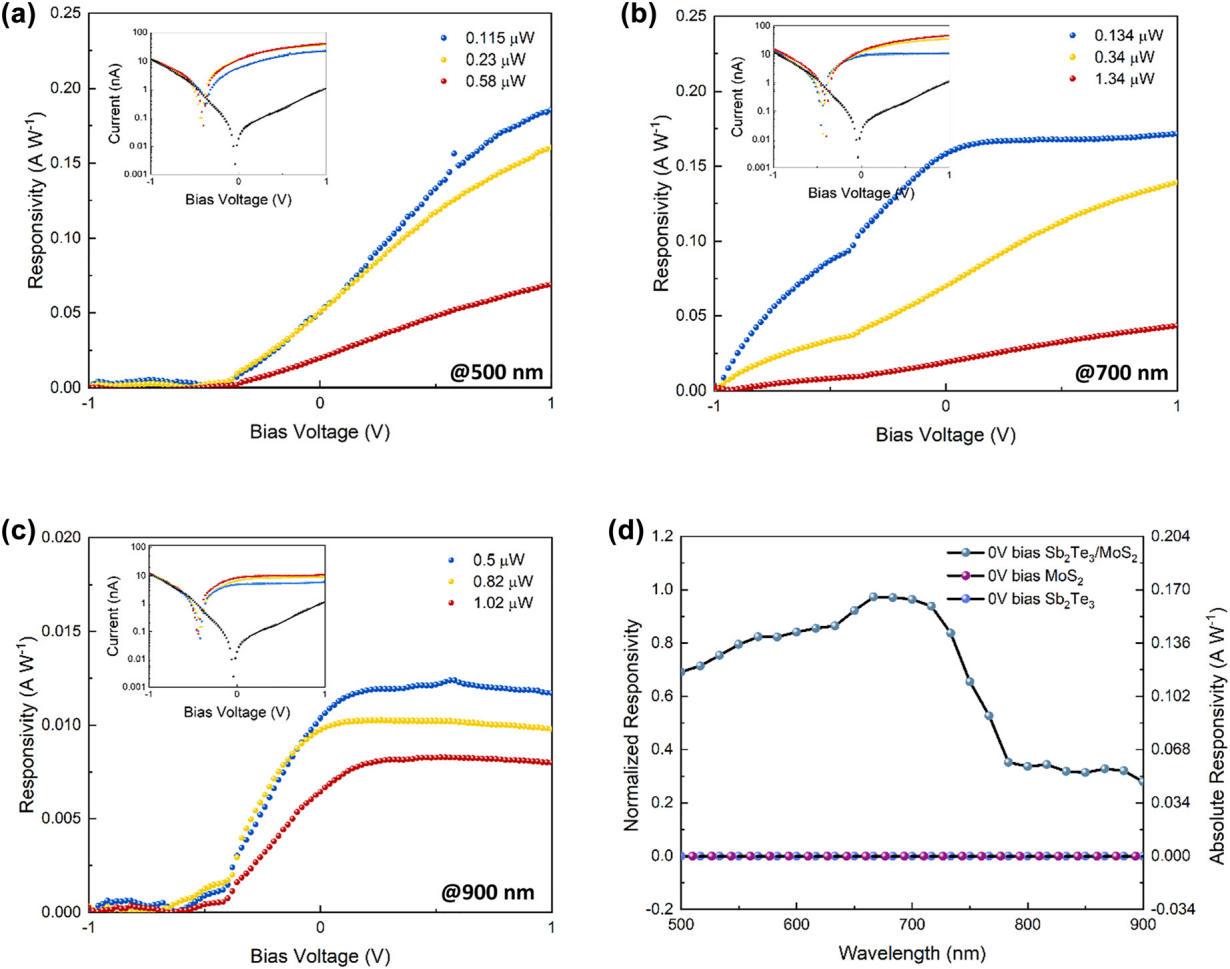
Electrical characterization of Sb_2_Te_3_/MoS_2_ PN junction heterostructure photodetector. (a), (b), (c) The measured photoresponsivity at different wavelengths, 500 nm, 700 nm, and 900 nm, under −1 to 1 V bias voltage. At zero bias, the photoresponsivity was clearly observed and increased proportionally to the optical power. (d) Normalized responsivity by sweeping the wavelength under 0 bias from 500 to 900 nm with a step size of 10 nm, which indicates a broad wavelength response.

Prior to the optoelectronic measurements, we characteristic the electrical performance of the MoS_2_/Sb_2_Te_3_ junction by applying a source-drain bias voltage *V*_sd_ from −1 to 1 V to evaluate the dark current and performance of the van der Waals PN heterojunction. As indicated by the black curve in the I-V measurement (inset of Figure 3a–c), the dark currents are stable and consistent. The dark current is as low as 2.38 pA at zero bias and 1.16 nA at 1 V bias, which benefits from the heterostructure design. To our best knowledge, this is the lowest dark current in a vdW heterojunction photodetector compared to other vdW heterojunction photodetectors [41–49]. At a negative bias, the dark current becomes significantly higher than at positive voltages. Furthermore, under on and off bias voltage *V*_sd_, the source-drain current, *I*_sd_ has a rectifying characteristic. This reveals that a vdW PN heterojunction is created at the interface of the stacked MoS_2_/Sb_2_Te_3_, since the rectifying effect is observed under different bias voltages. The diode’s on/off ratio, defined as the ratio between photogenerated current and dark current, is around 5.6 × 10^3^. At room temperature, the ultra-low dark current and high on/off ratio at zero bias enable the development of a highly sensitive self-powered photodetector. The power-dependent IV measurement of the PN heterojunction at the wavelength of 500 nm, 700 nm, and 900 nm has also been performed (inset of Figure 3a–c). At zero bias voltage, the heterojunction separates the photogenerated electro-hole pairs and introduces the photocurrent due to the built-in electric field formed at the MoS_2_/Sb_2_Te_3_ interface. The photocurrent *I*_photo_ increases with higher optical power illumination because electro-hole pairs generation rates increase. This demonstrates self-powered photodetection at various wavelengths (Figure 3a–c) with the insets in Figure 3a–c showing the measured *I*_photo_ and *I*_sc_ as a function of the optical power at various wavelengths. The *I*_photo_ increases.

Proportionally to the illuminated optical power, which gives evidence of the photovoltaic effect in the vdW PN heterojunction. The responsivity of the Sb_2_Te_3_/MoS_2_ PN junction-based heterostructure photodetector is measured from −1 V to +1 V at the wavelength of 500 nm, 700 nm, and 900 nm using the free-space optical setup. With a positive bias voltage, an external electric field is formed at the junction interface, increasing the separation efficiency of the photogenerated carriers and, therefore, the responsivity. At +1 V bias, the responsivity was amplified 1.5 to 3 times depending on the wavelength and optical power. The responsivity grows with the applied voltage and gradually saturates depending on the incident optical power. Under zero bias, the significant broadband photocurrent of the PN junction photodetector was observed within a wavelength range from 500 to 900 nm (Figure 3d). The highest responsivity under zero bias was 170 mA W^−1^ at 66 nm.

## 3 Conclusions

In conclusion, we proposed and experimentally demonstrated a self-powered, extremely sensitive photodetector based on a novel combination of a topological insulator and TMDs van der Waals PN heterojunction. Due to the current rectifying property of the PN heterojunction, the constructed device exhibits extremely low dark current and high responsivities. Under zero bias, the dark current of the device is 2.4 pA, and the responsivity of 170 mA W^−1^. By increasing the bias voltage to 1 V, the responsivity was magnified thrice due to the external field amplification. This outstanding performance of the proposed photodetector, with its novel material combination, paves the way for further research and applications based on other TMDs and Tis in optoelectronic applications. The PN heterojunction transfer can be easily integrated into waveguide systems, allowing for future applications in PICs.

## 4 Methods

### 4.1 Device fabrication

The Sb_2_Te_3_/MoS_2_ PN junction heterostructure is formed using 2D flakes exfoliated from the bulk crystals and transferred by the pick and drop transfer system on Si/SiO_2_ substrate. The electrical contact pads were formed using e-beam lithography (Raith Pioneer EBL), and electron beam evaporation was employed to deposit the Ti/Au (5 nm/50 nm) electrodes on the produced heterostructures. The lift-off was performed by acetone, then rinsing in isopropyl alcohol and nitrogen drying in RT (room temperature).

### 4.2 Device experimentation

The tunable (NKT SUPERCONTINUUM Compact) source and the source meter (Keithly 2600B) were used for electrical response measurements of Sb_2_Te_3_/MoS_2_ PN junction heterostructure devices. The laser beam was focused on the devices by an objective lens. The Raman was performed using a 532 nm laser source at room temperature.
